# Baseline Infection Burden and Cognitive Function in Elders with Essential Tremor

**DOI:** 10.5334/tohm.624

**Published:** 2021-05-11

**Authors:** Daniella Iglesias-Hernandez, Silvia Chapman, Keith Radler, Hollie Dowd, Edward D. Huey, Stephanie Cosentino, Elan D. Louis

**Affiliations:** 1 Department of Neurology, University of Texas Southwestern Medical Center, Dallas, Texas, US; 2 Department of Neurology, Vagelos College of Physicians and Surgeons, Columbia University, New York, US; 3 Taub Institute for Research on Alzheimer’s Disease and the Aging Brain, Vagelos College of Physicians and Surgeons, Columbia University, New York, US; 4 Movement Disorder Division, Department of Neurology, Yale School of Medicine, Yale University, New Haven, Connecticut, US; 5 Department of Psychiatry, Vagelos College of Physicians and Surgeons, Columbia University, New York, US

**Keywords:** essential tremor, epidemiology, risk factors, clinical, infection burden, self-reported questionnaires

## Abstract

**Background:**

Patients with essential tremor (ET) have an increased risk of cognitive impairment, yet little is known about the predictors of cognitive decline in these patients. Exposures to infectious agents throughout the lifespan may impact the later development of cognitive impairment. For example, high Infection exposure has been associated with lower cognitive performance in Alzheimer’s and Parkinson’s disease. However, this predictor has not been examined in ET.

**Objectives:**

To determine whether a higher baseline infection burden is associated with worse cognitive performance at baseline and greater cognitive decline over time in an ET cohort.

**Method/Design:**

160 elderly non-demented ET participants (80.0 ± 9.5 years) underwent an extensive cognitive evaluation at three time points. At baseline, participants completed an infection burden questionnaire (t-IBQ) that elicited information on previous exposure to infectious agents and number of episodes per disease. Analysis of covariance and generalized estimated equations (GEEs) were used.

**Results:**

Overall, infection burden was not associated baseline cognitive performance. Adjusted GEE models for repeated measures yielded a significant time interaction between moderate infection burden at baseline and better performance in the attention domain over time (*p* = 0.013). Previous history of rubella was associated with faster rate of decline in visuospatial performance (*p* = 0.046).

**Conclusion:**

The data were mixed. Moderate self-reported infection burden was associated with better attention performance over time. Self-reported history of rubella infection was related to lower visuospatial performance over time in this cohort. Follow-up studies with additional design elements would be of value.

## Introduction

Essential tremor (ET) is one of the most common movement disorders, with a worldwide prevalence of 4.6% in adults age 65 and older [1]. ET has traditionally been characterized by its motor features [2]. However, recent evidence has shown that ET is a multidimensional disorder with non-motor (e.g., cognitive) features as well [3]. Indeed, patients with ET appear to have an increased odds or risk of developing mild cognitive impairment (MCI) and dementia [4,5,6,7]. While the characterization of cognitive deficits in ET remains ongoing, surprisingly little is known about the predictors of cognitive impairment and decline in these patients [4,8]. Hence, the epidemiology of cognitive decline in ET is largely unexplored.

Exposures throughout the lifespan may impact the later development and progression of cognitive impairment over time. These exposures may range from toxicological to traumatic to infections [9,10,11]. Infection burden has been studied as a predictor of cognitive decline in several settings and with many different approaches [12,13]. Several different mechanisms by which infection burden could influence cognitive impairment have been hypothesized. First, specific infectious agents may influence the accumulation of neuropathological changes associated with dementia [14,15]. For example, herpes simplex virus (HSV) and respiratory syncytial virus (RSV) could promote the aggregation of amyloid β-peptide, a major component of amyloid plaques in Alzheimer’s disease (AD) [14]. Second, infectious epitopes can trigger chronic inflammation in the central nervous system, potentially predisposing for neuropsychiatric disorders [16,17]. In support of these hypothesis, high immunoglobulin titers for several different viruses including HSV, RSV, hepatitis B virus and cytomegalovirus (CMV) have been associated with poor cognitive performance in AD and Parkinson’s disease (PD) [17,18]. Additional studies have demonstrated that greater infection burden was associated with worse global cognition at baseline and decreased memory performance over time in a multiethnic cohort [19,20,21].

As noted above, infectious exposures have been examined in the context of several neurological disorders, with an emphasis on cognitive performance in diseases related to ET such as AD and PD [22,23,24]. To our knowledge, however, baseline infection burden has not been examined as a risk factor for cognitive decline in ET. We hypothesize that a higher overall baseline infection burden would be associated with lower cognitive performance at baseline and would predict greater cognitive decline over time in our ET cohort. We also explored the effects of certain specific infectious agents that have been implicated as associated with cognitive impairment in other disorders.

## Methods

### Study Design

The Clinical-Pathological Study of Cognitive Impairment in ET (COGNET) is an ongoing, prospective, longitudinal study of cognition and its neuropathological correlates in an elderly ET cohort. Eligible participants met each of the following criteria: (1) diagnosis of ET in the absence of other movement disorders, (2) willingness to become a brain donor, (3) willingness to participate in extensive cognitive testing every 1.5 years, and (4) no previous brain surgery for ET. Between 2014 and 2019, 186 participants were interviewed by trained research assistants at three different time points: baseline (T1), 18 months after baseline (T2), and 36 months after baseline (T3). Demographic and clinical data were collected at each interview. During each interview, a neuropsychological test battery was administered over two consecutive days. A videotaped neurological evaluation, followed by a tremor rating by a senior movement disorders neurologist (E.D.L.), resulted in a total tremor score (0–36) [25,26], and the final diagnosis of ET was assigned using valid and reliable criteria [27]. The Internal Review Boards of University of Texas Southwestern Medical Center and Columbia University approved the study protocol and each participant provided informed, written consent during the in-person visit.

### Neurocognitive Evaluation

The neuropsychological battery was designed to measure performance in overall cognition and five cognitive domains: memory, executive function, attention, language, and visuospatial function. As described previously, the test battery was specifically designed for the ET cohort, as it excluded tests for which scores rely on the speed or accuracy of motor responses [28].

For each interval, the research team conducted an informant’s interview with a designated family member or close friend. The informant answered several questionnaires related to the participant’s daily life and level of involvement with their household and community [28].

After every interview, Clinical Dementia Rating Score (CDR) (0 = no dementia, 0.5 = questionable dementia, 1 = mild dementia, 2 = moderate dementia, and 3 = severe dementia) [29] and cognitive diagnosis (*normal cognition* (*ET-NC*), *mild cognitive impairment* (*ET-MCI*), or *dementia* (*ET-D*)) were assigned to participants during a consensus conference. A neuropsychologist (S.C.) and geriatric psychiatrist (E.D.H.) reviewed CDR scores assigned by the research assistant based on examiner and informant interview, and assigned diagnoses based on CDR score and neuropsychological testing [30]. Raw cognitive test scores were standardized using the mean and standard deviation of the ET-NC group.

### Infection Burden Questionnaire

Twenty-four common infectious agents were itemized in 25 questions (Supplementary Figure 1). The viral infections section assessed: Influenza virus, Varicella zoster (*Alphaherpesviridae*) (reported in the questionnaire as either shingles or chickenpox), Rhinovirus (*Picornaviridae enterovirus*), Measles virus (*Paramyxoviriade family*), Mumps virus *(Paramyxoviriade family)*, Rubella virus (*Togaviridae*), Hepatitis A (Picornaviridae), Hepatitis B (*Hepadnaviridae*), Hepatitis C (*Flaviviridae*), Cytomegalovirus *(Herpesviridae)*, Poliovirus (*Picornaviridae*), Ebstein-Barr virus (*Herpesviridae)*, Herpes Simplex Virus type 1 (HSV1) and type 2 (HSV-2) (*Herpesviridae*), and Human Immunodeficiency Virus (HIV) (*Retroviridae*). For bacterial agents, the following microorganisms were included: *Streptococcus pyogenes, Borrelia burgdorferi, Clostridium tetani, Vibrio cholera, Yersinia pestis, Mycobacterium tuberculosis, Treponema pallidum, Chlamydia trachomatis*, and *Neisseria gonorrhoeae* (Supplementary Figure 1).

Research assistants administered the questionnaire at baseline and employed non-scientific terms to describe the infectious diseases following published recommendations [31]. For each question *(“have you ever had this infection?”)*, the participant could answer “yes” or “no” to the questions, and 1 point was allotted for every “yes”. A third response could be “I don’t know” and that answer received 0 points when calculating the index. Raw infection burden (r-IBQ) was computed by adding the number of times the participant answered “yes” and possible values ranged from 0 to 24. Next, the participants indicated how many times in their lifetime they had had each infection and total infection burden (t-IBQ) was calculated by adding the total frequencies (except for Rhinovirus or common cold, which was very frequent and would have dwarfed other data). The possible values could range from 0 to infinity.

### Geriatric Depression Scale and Physical Activity Scale of the Elderly

Due to the potential association between physical activity, depression and cognitive impairment, two additional questionnaires administered at baseline were included in the statistical analyses [32,33]. Depression was measured using the Geriatric Depression Scale (GDS). The instrument relies on self-report and the values range from 0 to 30, with higher values indicating greater depressive symptoms [34]. Second, physical activity was measured using the Physical Activity Scale for the Elderly (PASE), a valid and reliable measure of leisure time, household, and work-related physical activity. The questionnaire is based on 10 items and scores can range from 0 to 400, although in some cases higher values can be registered [34,35]. Higher scores indicate more physical activity.

### Final Sample

Initially, the study enrolled 243 participants. A total of 83 cases were excluded from the analysis according to the following criteria: diagnosis of MCI or dementia at baseline (n = 11); diagnosis of ET with dystonic or parkinsonian features (n = 38); only completed one interview (n = 34). Of the remaining 160 participants, 120 participants fully completed the IBQ questionnaire and 40 did not due to time constraints during the interviews. We analyzed the two groups to evaluate for a possible no-response bias. The 40 participants who did not complete the questionnaire had a mean age of 79 years (SD = 9.6), a mean education level of 15 years (SD = 2.5), mean tremor duration of 40 years (SD = 21.0) and 30 (75.0%) were female. The 120 participants that answered the questionnaire had similar characteristics: a mean age of 77 years (SD = 39.0), mean education level of 16 years (SD = 2.6), mean tremor duration of 36 years (SD = 23.3) and 60 (50%) were female. The gender difference was significant (chi-square = 4.73, *p* = 0.03). For the statistical analyses we included only the participants that fully completed the questionnaire (n = 120).

### Statistical Analyses

Variables at baseline were described using mean and standard deviation if continuous, and frequencies and percentages if categorical. Standardized z scores were assigned for each participant’s cognitive domains applying the methodology described above. Furthermore, t-IBQ was transformed to a logarithmic scale due to the non-normal distribution of the data. The r-IBQ were stratified into two categories: low and high infection burden. The t-IBQ had a wider range and was stratified into three categories: low, moderate, and high infection burden. One-way ANOVA was used to examine significant differences between the means in age, years of education, number of medications, PASE, GDS, and cognitive domains z scores across the three levels of t-IBQ. We implemented one way analysis of covariance (ANCOVA) to determine the potential association between infectious burden at baseline and z scores of cognitive domains (global, memory, executive function, attention, language and visuospatial) while controlling for the variables previously described.

For repeated measures, generalized estimating equations (GEEs) were used to assess the effect of baseline t-IBQ on performance for each cognitive domain over time. The role as a predictor between infection burden at baseline and z scores of each cognitive domain was evaluated through the time interaction of the model. Initial unadjusted models were conducted to observe the nature of the interactions and subsequent adjusted models included the following covariates at baseline as potential confounders: age, gender, years of education, total number of prescription medications, PASE, and GDS.

Self-reported past infections of Rubella, Measles, and HSV-1 were evaluated as potential predictors of cognitive decline in individual GEE models. These three microorganisms have been extensively associated with neuropathological changes in the central nervous system that might affect higher cognitive functions [36,37,38]. Since using an index that combines numerous different infectious agents might mask the effect of certain viruses in the outcome, this analysis was deemed necessary [39,40]. The predictors were dichotomized as “0” if no history of infection was mentioned or “1” if the participant had had the disease at least once. Unadjusted GEE models were followed by adjusted GEE models to control for potential confounding effects. Data analysis was performed using IBM SPSS v. 26.

## Results

The mean age of our participants was 80.0 ± 9.5 years (range = 57–97 years) (***Table 1***). The score for r-IBQ ranged from 2 to 9 (mean = 5.9, SD = 1.89), and the t-IBQ ranged from 1 to 369 (mean = 73.8, SD = 65.2) (***Table 1***).

**Table 1:** Baseline features of 120 ET participants.


	MEAN ± STANDARD DEVIATION OR N (%)

Age (years)	80.0 ± 9.5

Gender (female)	73 (60.8)

Education (years)	15.7 ± 2.6

Number of prescription medications	5.6 ± 4.1

PASE score	106.9 ± 74.0

GDS score	6.5 ± 4.6

Cognitive Z scores	

Overall	0.01 ± 0.53

Memory	–0.02 ± 0.90

Executive Function	0.05 ± 0.64

Attention	–0.22 ± 0.77

Language	0.05 ± 0.53

Visuospatial	0.47 ± 0.67

Rubella in childhood	29 (23.2)

Raw Infection burden (r-IBQ)	5.9 ± 1.8

Categorical raw infection burden (r-IBQ):	

Low (0–4)	49 (40.8)

High (5–9)	71 (59.2)

Total infection burden (t-IBQ)	73.8 ± 65.2

Categorical total infection burden (t-IBQ):	

Low (1–37)	40 (33.3)

Moderate (38–87)	39 (32.5)

High (≥88)	39 (32.5)


*Note*: GDS = Geriatric Depressive Symptoms Scale, PASE = Physical Activity Scale of the Elderly, bolded numbers indicate significant p values (p < 0.05).

Comparison of the means showed significant differences in overall cognition (F = 3.18, *p* = 0.046) and visuospatial function (F = 3.25, *p* = 0.04) across the three levels of t-IBQ. Participants with low infection burden had lower z scores in global cognition (–0.24 ± 0.74) and visuospatial (0.41 ± 0.66) domains, suggesting worse cognitive performance as compared to participants in the moderate and high infection burden categories (***Table 2***). However, ANCOVA did not reveal any significant associations between baseline t-IBQ and z scores for each cognitive domain after controlling for the following baseline covariates: age, gender, years of education, medications, PASE and GDS (***Table 3***).

**Table 2:** Demographic and clinical data across strata of low, moderate and high infection burden (t-IBQ).


	MEAN (STANDARD DEVIATION)	F	P-VALUE

Age (years)		0.44	0.65

Low t-IBQ	78.9 (9.1)		

Moderate t-IBQ	77.0 (9.3)		

High t-IBQ	77.6 (9.3)		

Education (years)		0.63	0.53

Low t-IBQ	15.8 (2.5)		

Moderate t-IBQ	15.1 (6.1)		

High t-IBQ	15.8 (2.7)		

Number of prescription medications		1.45	0.24

Low t-IBQ	5.5 (3.1)		

Moderate t-IBQ	5.3 (3.8)		

High t-IBQ	6.1 (4.4)		

PASE score		1.44	0.24

Low t-IBQ	113.8 (78.1)		

Moderate t-IBQ	96.7 (78.4)		

High t-IBQ	108.1 (68.0)		

GDS score		2.23	0.11

Low t-IBQ	5.5 (4.3)		

Moderate t-IBQ	6.6 (5.4)		

High t-IBQ	6.0 (5.0)		

Cognitive z scores at baseline			

Overall		3.18	**0.046**

Low t-IBQ	–0.24 (0.74)		

Moderate t-IBQ	0.08 (0.84)		

High t-IBQ	0.22 (0.51)		

Memory		2.38	0.10

Low t-IBQ	0.06 (0.72)		

Moderate t-IBQ	0.43 (0.73)		

High t-IBQ	0.27 (0.27)		

Executive Function		0.83	0.44

Low t-IBQ	0.15 (0.58)		

Moderate t-IBQ	0.31 (0.40)		

High t-IBQ	.26 (00.48)		

Attention		0.56	0.57

Low t-IBQ	–0.21 (0.68)		

Moderate t-IBQ	–0.03 (0.79)		

High t-IBQ	–0.05 (0.73)		

Language		0.39	0.69

Low t-IBQ	.012 (0.45)		

Moderate t-IBQ	0.18 (0.47)		

High t-IBQ	0.60 (0.63)		

Visuospatial		3.25	**0.04**

Low t-IBQ	0.41 (0.66)		

Moderate t-IBQ	0.54 (0.63)		

High t-IBQ	0.80 (0.58)		


*Note*: GDS = Geriatric Depressive Symptoms Scale, PASE = Physical Activity Scale of the Elderly, bolded numbers indicate significant p values (p < 0.05).

**Table 3:** Analysis of covariance between baseline total infectious burden (t-IBQ) and baseline global cognition, memory, executive function, attention, language and visual spatial domains.


	F	MEAN SQUARE	P-VALUE

**Global Cognition**			

Age	0.071	0.02	0.790

Male vs. female	0.015	0.01	0.930

Years of education	0.225	0.06	0.636

Medications	0.795	0.20	0.375

PASE score	4.175	1.06	**0.044**

GDS score	0.245	0.07	0.622

Total infection burden (t-IBQ categorical)	1.442	3.65	0.242

**Memory**			

Age	0.038	0.029	0.846

Male vs. female	0.065	0.050	0.800

Years of education	0.365	0.283	0.547

Medications	0.046	1.902	0.121

PASE score	2.454	0.024	0.835

GDS score	0.043	0.036	0.830

Total infection burden (t-IBQ categorical)	2.180	1.690	0.119

**Executive Function**			

Age	0.215	0.097	0.644

Male vs. female	0.369	0.166	0.545

Years of education	0.015	0.007	0.903

Medications	0.229	0.103	0.633

PASE score	2.824	1.271	0.096

GDS score	0.706	0.318	0.403

Total infection burden (t-IBQ categorical)	0.660	0.297	0.519

**Attention**			

Age	1.122	0.595	0.292

Male vs. female	0.404	0.214	0.527

Years of education	0.489	0.259	0.486

Medications	0.147	0.078	**0.017**

PASE score	5.855	3.104	0.690

GDS score	0.160	0.085	0.703

Total infection burden (t-IBQ categorical)	0.147	1.264	0.098

**Language**			

Age	2.088	0.592	0.152

Male vs. female	0.011	0.003	0.918

Years of education	0.064	0.018	0.801

Medications	1.554	0.441	0.435

PASE score	0.616	0.175	0.490

GDS score	0.481	0.136	0.216

Total infection burden (t-IBQ categorical)	0.319	0.090	0.728

**Visuospatial**			

Age	0.075	0.036	0.783

Male vs. female	0.001	0.001	0.971

Years of education	3.402	1.610	0.068

Medications	1.317	0.623	0.254

PASE score	0.433	0.205	0.512

GDS score	0.055	0.026	0.815

Total infection burden (t-IBQ categorical)	0.090	0.042	0.914


*Note*: GDS = Geriatric Depressive Symptoms Scale, PASE = Physical Activity Scale of the Elderly, bolded numbers indicate significant p values (p < 0.05).

The longitudinal analysis included 120 participants for whom 120 observations were recorded at baseline, 120 at T2 and 110 at T3, for a total of 350 repeated measures used in the GEE models. Initial unadjusted models showed no significant association between categorized t-IBQ and cognitive outcomes at baseline. In these unadjusted models, the association between t-IBQ at baseline and cognitive z scores by time interaction was not significant in any of the levels of the variable (see ***Table 4***). Similarly, the adjusted models yielded no significant associations between t-IBQ and cognitive z scores at baseline. However, there was a significant time interaction in the attention domain where moderate t-IBQ predicted better performance over time (b = 0.01, *p* = 0.013). (***Table 4***).

**Table 4:** Generalized estimated equations of global cognition, memory, executive function, attention, language and visual spatial performance predicted by total infection burden (t-IBQ).


	B (SE)	P-VALUE

**Global Cognition**		

***Unadjusted model main effects***:		

Time from baseline (months)	0.00 (0.00)	0.901

Baseline total infection burden		

Moderate (38–87)	0.02 (0.12)	0.840

High (≥88)	0.06 (0.14)	0.670

***Unadjusted model time interaction***:		

Time × Baseline total infection burden interaction		

Moderate (38–87)	0.00 (0.00)	0.519

High (≥88)	0.00 (0.01)	0.946

***Adjusted model main effects***:		

Baseline age	–0.03 (0.05)	**<0.001**

Male vs. female	0.05 (0.09)	0.618

Baseline education	0.03 (0.02)	0.167

Medications	–0.03 (0.01)	0.054

PASE score	0.00 (0.00)	0.109

GDS score	–0.02 (0.01)	0.667

Time from baseline (months)	0.00 (0.00)	0.783

Baseline total infection burden		

Moderate (38–87)	0.02 (0.09)	0.810

High (≥88)	0.01 (0.10)	0.904

***Adjusted model with time interaction***:		

Time × Baseline total infection burden interaction		

Moderate (38–87)	0.02 (0.09)	0.375

High (≥88)	0.00 (0.01)	0.919

**Memory**	**B (se)**	**p-value**

***Unadjusted model main effects***:		

Time from baseline (months)	0.01 (0.00)	**0.018**

Baseline total infection burden		

Moderate (38–87)	0.14 (0.19)	0.459

High (≥88)	–0.01 (0.17)	0.939

***Unadjusted model with time interaction***:		

Time × baseline total infection burden interaction		

Moderate (38–87)	–0.03 (0.00)	0.439

High (≥ 88)	–0.03 (0.01)	0.569

***Adjusted model main effects***:		

Baseline age	–0.03 (0.01)	**<0.001**

Male vs. female	–0.32 (0.14)	**0.024**

Baseline education	0.08 (0.03)	**0.026**

Number of medications	–0.02 (0.02)	0.389

PASE score	0.00 (0.00)	0.235

GDS score	0.01 (0.02)	0.753

Time from baseline (months)	0.01 (0.00)	0.**008**

Baseline total infection burden		

Moderate (38–87)	0.11 (0.16)	0.620

High (≥88)	–0.14 (0.14)	0.340

***Adjusted model with time interaction***:		

Time × Baseline total infection burden interaction		

Moderate (38–87)	0.00 (0.00)	0.335

High (≥88)	–0.01 (0.01)	0.561

**Executive Function**	**B (se)**	**p-value**

***Unadjusted model main effects***:		

Time from baseline (months)	–0.01 (0.00)	**0.003**

Baseline total infection burden		

Moderate (38–87)	–0.13 (0.19)	0.497

High (≥ 88)	0.08 (0.19)	0.668

***Unadjusted model with time interaction***:		

Time × Baseline total infection burden interaction		

Moderate (38–87)	0.01 (0.01)	0.054

High (≥88)	–0.01 (0.00)	0.289

***Adjusted model main effects***:		

Baseline age	–0.03 (0.01)	**<0.001**

Male vs. female	0.117 (0.10)	0.252

Baseline education	0.04 (0.02)	0.066

Number of medications	–0.05 (0.02)	**0.006**

PASE score	0.00 (0.00)	0.349

GDS score	0.00 (0.01)	0.734

Time from baseline (months)	–0.01 (0.00)	0.418

Baseline total infection burden		

Moderate (38–87)	0.01 (0.04)	0.188

High (≥88)	–0.02 (0.11)	0.786

***Adjusted model with time interaction***:		

Time × Baseline total infection burden interaction		

Moderate (38–87)	0.01 (0.00)	0.188

High (≥88)	–0.01 (0.01)	0.786

**Attention**	**B (se)**	**p-value**

***Unadjusted model main effects***:	0.00 (0.00)	**0.003**

Time from baseline (months)		

Baseline total infection burden		

Moderate (38–87)	–1.28 (0.19)	0.497

High (≥88)	0.08 (.019)	0.668

***Unadjusted model with time interaction***:		

Time × Baseline total infection burden interaction		

Moderate (38–87)	0.01 (0.00)	0.054

High (≥88)	0.01 (0.01)	0.289

***Adjusted model main effects***:		

Baseline age	–0.04 (0.01)	**<0.001**

Male vs. female	0.02 (0.12)	0.999

Baseline education	0.01 (0.02)	0.769

Number of medications	–0.05 (0.01)	**<0.001**

PASE score	0.00 (0.00)	0.326

GDS score	–0.00 (0.01)	0.879

Time from baseline (months)	–0.01 (0.00)	**0.040**

Baseline total infection burden		

Moderate (38–87)	–0.13 (.13)	0.255

High (≥88)	0.04 (.12)	0.842

***Adjusted model with time interaction***:		

Time × Baseline total infection burden interaction		

Moderate (38–87)	0.01 (.00)	**0.013**

High (≥88)	0.01 (.01)	0.134

**Language**	**B (se)**	**P value**

***Unadjusted model main effects***:		

Time from baseline (months)	–0.01 (0.00)	0.217

Baseline total infection burden		

Moderate (38–87)	0.01 (0.01)	0.852

High (≥88)	0.04 (0.01)	0.611

***Unadjusted model with time interaction***:		

Time × Baseline total infection burden interaction		

Moderate (38–87)	0.01 (0.01)	0.852

High (≥88)	0.01 (0.01)	0.611

***Adjusted model main effects***:		

Baseline age	–0.03 (0.01)	**0.001**

Male vs. female	0.44 (0.15)	**0.003**

Baseline education	0.02 (0.04)	0.628

Number of medications	–0.01 (0.02)	0.875

PASE score	0.00 (0.00)	0.789

GDS score	–0.01 (0.02)	0.799

Time from baseline (months)	0.04 (0.00)	0.374

Baseline total infection burden		

Moderate (38–87)	0.37 (0.01)	0.863

High (≥88)	–0.18 (0.27)	0.513

***Adjusted model with time interaction***:		

Time × Baseline total infection burden		

Moderate (38–87)	0.00 (0.01)	0.590

High (≥88)	0.00 (0.01)	0.554

**Visuospatial**	**B (se)**	**p value**

***Unadjusted model main effects***:		

Time from baseline (months)	0.00 (0.00)	0.929

Baseline total infection burden		

Moderate (38–87)	0.83 (0.16)	0.596

High (≥88)	0.25 (0.19)	0.163

***Unadjusted model with time interaction***:		

Time × Baseline total infection burden		

Moderate (38–87)	–0.02 (0.04)	0.604

High (≥88)	–0.01 (0.01)	0.252

***Adjusted model main effects***:		

Baseline age	–0.03 (0.00)	**<0.001**

Male vs. female	0.06 (0.12)	0.601

Baseline education	0.01 (0.03)	0.762

Number of medications	–0.01 (0.02)	0.833

PASE score	0.01 (0.00)	0.091

GDS score	–0.02 (0.01)	0.174

Time from baseline (months)	–0.01 (0.00)	0.537

Baseline total infection burden		

Moderate (38–87)	0.10 (0.14)	0.455

High (≥88)	0.23 (0.14)	0.116

***Adjusted model with time interaction***:		

Time × Baseline total infection burden		

Moderate (38–87)	–0.01 (0.05)	0.964

High (≥88)	–0.01 (0.01)	0.584


*Note*: GDS = Geriatric Depressive Symptoms Scale, PASE = Physical Activity Scale of the Elderly, bolded numbers indicate significant p values (p < 0.05).

Similar adjusted and unadjusted models with r-IBQ as potential predictor yielded no significant associations (all p > 0.05) (data not shown).

Subsequent GEE models were performed with individual infectious agents (Rubella, Measles and HSV-1), as discussed in the Methods section. For the unadjusted models, rubella was the only agent significantly associated with the time trend of the visuospatial z scores (B = –0.01, *p* = 0.014). In adjusted models, the same time interaction was observed (B = –0.01, *p* = 0.034) indicating that previous rubella infection was associated with a decrease of 0.01 in the time trend for visuospatial z scores (***Table 5***).

**Table 5:** Generalized estimated equations of visual spatial performance predicted by previous Rubella infection.


VISUOSPATIAL	B (SE)	p-value

***Unadjusted model main effects***:		

Time from baseline (months)	–0.01 (0.00)	0.33

Baseline Rubella in childhood	0.08 (0.17)	0.66

***Unadjusted model with time interaction***:		

Time × Rubella in childhood	–0.01 (0.01)	**0.014**

***Adjusted model main effects***:		

Time from baseline (months)	0.00 (0.00)	0.935

Baseline Rubella in childhood	0.08 (0.12)	0.546

Baseline age	–0.04 (0.01)	**<0.001**

Male vs. female	0.03 (0.12)	0.858

Baseline education	0.01 (0.02)	0.500

Number of medications	0.01 (0.01)	0.969

PASE score	0.00 (0.00)	0.105

GDS score	–0.02 (0.01)	0.155

***Adjusted model with time interaction***:		

Time × Rubella in childhood	–0.01 (0.01)	**0.034**


*Note*: GDS = Geriatric Depressive Symptoms Scale, PASE = Physical Activity Scale of the Elderly, bolded numbers indicate significant p values (p < 0.05).

## Discussion

In previous studies of cognitively normal adults, high infection burden has been associated with lower global cognition [19,20]. The literature also shows that higher seropositivities have been associated with lower mini-mental state examination (MMSE) scores in a cohort of AD adults [18]. In 2005, Dunn et al. established that diagnosis of dementia in an elderly cohort was associated with a history of two or more infections in the four years preceding the diagnosis [40]. Additional evidence spans the last two decades with multiple publications aiming to identify the role of infectious diseases in cognitive decline [18,39,41].

The COGNET study is in a unique position to explore the impact of infection burden in ET because of the detailed, prospective, longitudinal cognitive evaluation. Overall, we only found an association between moderate infectious burden and better performance over time in the attention domain. Ecological studies have found similar results where childhood infectious diseases have been associated with both positive and negative cognitive outcomes in adulthood [42,43]. A population based study of healthy adults over 65 also determined that late-life MMSE scores improved as the number of reported childhood diseases (chickenpox, measles and mumps) increased [44]. Nevertheless, the mechanisms for possible positive outcomes in cognition are not clear [42,45]. The evidence in the literature must be treated cautiously due to potential unaccounted confounding as well as the ecological fallacy [46,47].

At the same time, previous history of rubella infection might predict lower cognitive performance in visuospatial function over time. These results should be confirmed by further studies.

Rubella has been extensively studied because of its effect in pregnancy and potentially fatal complications such as multiphasic acute disseminated encephalomyelitis [48]. In both congenital and childhood postnatal infection, development of progressive neurologic deterioration often manifests as prominent cognitive impairment, seizures, cerebellar degeneration, and dementia [49]. However, subtle changes in cognition over time have not been described in cohorts with prior rubella infection.

**Figure 1:**
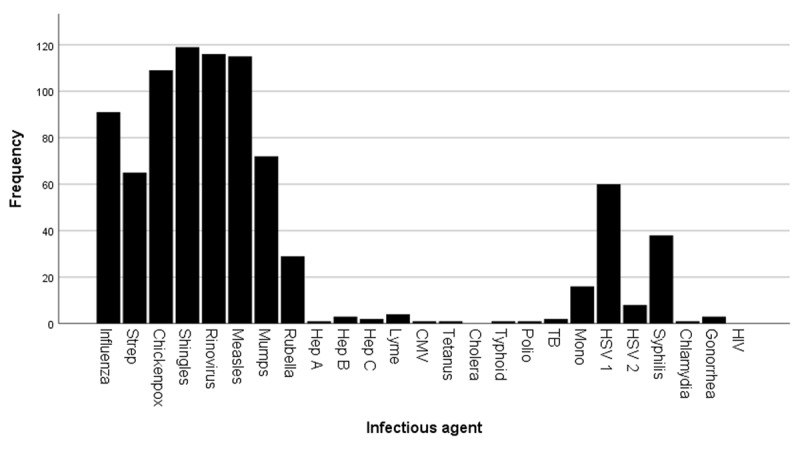
Frequency of positive answers by infection agent. For each item, the 120 participants answered “yes” or “no” according to their previous medical history. *Note*: Strep = Streptococcus, Hep = Hepatitis, CMV = Cytomegalovirus, Polio = Poliomyelitis, TB = Tuberculosis, Mono = Mononucleosis, HSV = Herpes simplex virus, HIV = human immunodeficiency virus.

An important factor to consider is the age of the cohort and the prevalence of certain infections in the last century. The mean age of our participants was 80 years and common childhood diseases such as measles and rubella were more prevalent before the MMR vaccine was distributed in the United States in 1963 [50]. Before nationwide vaccination, more than 90% of the worldwide population had been infected with measles between 10 and 15 years of age [50]. This high prevalence is reflected in the results we report, as 96% (n = 115) of the participants answered “yes” when asked about previous infections with measles (***Figure 1***). Therefore, assessing an interaction becomes challenging when the majority of the cohort has been exposed to said agent.

Another limitation was the use of a self-reported questionnaire to measure infection burden. The instrument relies heavily on the memory of participants, increasing the possibility of recall bias. This is the main reason why participants diagnosed with MCI or dementia at baseline were excluded from the analyses [51]. Additional limitations of this instrument include the level of knowledge needed to identify several infectious diseases increasing the possibility of underreport [31]. Hence, the literature favors alternative approaches to measure infection burden such as antibody titers and disability adjusted life years (DALY) [21,52,53,54,55]. Nevertheless, self-report questionnaires are considered reliable and valid and are frequently used in epidemiological studies to complement objective data [48,56,57]. This analysis is in many ways a preliminary, hypothesis-generating one, and future studies, more narrowly focused, should explore the use of such titers. Furthermore, additional approaches, such as the use of medical records, national databases and immunoglobulin titers could complement the information gathered through clinical questionnaires [56]. One other potential limitation is that we found that the 120 participants who answered the questionnaire were less likely to be female than the 40 who did not. It is unlikely, though, that this difference affected our results; furthermore, we adjusted for gender in our analyses.

These manuscript joins a growing number of studies focused on the association between infections and cognitive function and, to our knowledge, are the only such data for ET. Moderate infectious burden might be associated to better performance over time in the attention domain. On the other hand, Rubella could be involved in this cohort’s lower performance in the visuospatial domain overtime. The research group encourages further analyses to explore the nature of the observed interactions.

## Additional File

The additional file for this article can be found as follows:

10.5334/tohm.624.s1 Supplementary Figure 1. Infection Burden Questionnaire administered at baseline.
