# Supplementary material for: Baseline Infection Burden and Cognitive Function in Elders with Essential Tremor
Source: Tremor Other Hyperkinet Mov (N Y). 2021 May 11;11:16. doi: 10.5334/tohm.624 (PMC8121006; doi:10.5334/tohm.624)
Supplement: Supplementary Figure 1. — Infection Burden Questionnaire administered at baseline. [file tohm-11-1-624-s1.pdf]

Supplementary Figure 1: Infection Burden Questionnaire administered at baseline

Infectious Burden Questionnaire

For the following list of infections, please indicate whether you have ever had the infection at any point in the last 18 months. Circle Yes, No, or DNK (I don't know). If Yes, please indicate the approximate number of times you had had this infection throughout your lifetime.

Have you ever had:

1. Influenza ("Flu")	YES	NO	DNK	How many times?: ________
2. Strep Throat:	YES	NO	DNK	How many times?: ________
3. Chicken Pox:	YES	NO	DNK	How many times?: ________
4. Shingles:	YES	NO	DNK	How many times?: ________
5. Common Cold:	YES	NO	DNK	How many times?: ________
6. Measles:	YES	NO	DNK	How many times?: ________
7. Mumps:	YES	NO	DNK	How many times?: ________
8. Rubella:	YES	NO	DNK	How many times?: ________
9. Hepatitis A:	YES	NO	DNK	How many times?: ________
10. Hepatitis B:	YES	NO	DNK	How many times?: ________
11. Hepatitis C:	YES	NO	DNK	How many times?: ________
12. Lyme Disease:	YES	NO	DNK	How many times?: ________
13. Cytomegalovirus:	YES	NO	DNK	How many times?: ________

14. Tetanus:	YES	NO	DNK	How many times?: ________
15. Cholera:	YES	NO	DNK	How many times?: ________
16. Typhoid Fever:	YES	NO	DNK	How many times?: ________
17. Poliomyelitis	YES	NO	DNK	How many times?: ________
18. Tuberculosis:	YES	NO	DNK	How many times?: ________
19. Mononucleosis:	YES	NO	DNK	How many times?: ________
20. Cold Sores:	YES	NO	DNK	How many times?: ________
21. Genital Herpes:	YES	NO	DNK	How many times?: ________
22. Syphilis:	YES	NO	DNK	How many times?: ________
23. Chlamydia:	YES	NO	DNK	How many times?: ________
24. Gonorrhea:	YES	NO	DNK	How many times?: ________
25. HIV:	YES	NO	DNK	How many times?: ________
